# Phase I First-in-Human Dose Escalation Study of the oral SF3B1 modulator H3B-8800 in myeloid neoplasms

**DOI:** 10.1038/s41375-021-01328-9

**Published:** 2021-06-25

**Authors:** David P. Steensma, Martin Wermke, Virginia M. Klimek, Peter L. Greenberg, Patricia Font, Rami S. Komrokji, Jay Yang, Andrew M. Brunner, Hetty E. Carraway, Lionel Ades, Aref Al-Kali, Juan M. Alonso-Dominguez, Ana Alfonso-Piérola, Catherine C. Coombs, H. Joachim Deeg, Ian Flinn, James M. Foran, Guillermo Garcia-Manero, Michael B. Maris, Malgorzata McMasters, Jean-Baptiste Micol, Jaime Perez De Oteyza, Felicitas Thol, Eunice S. Wang, Justin M. Watts, Justin Taylor, Richard Stone, Vikram Gourineni, Alyssa J. Marino, Huilan Yao, Benoit Destenaves, Xiaobin Yuan, Kun Yu, Sara Dar, Lernik Ohanjanian, Keisuke Kuida, Jianjun Xiao, Catherine Scholz, Antonio Gualberto, Uwe Platzbecker

**Affiliations:** 1 Dana–Farber Cancer Institute, Boston, MA USA; 2 University Hospital Dresden, Dresden, Germany; 3 Memorial Sloan Kettering Cancer Center, New York, NY USA; 4 Stanford University Medical Center, Stanford, CA USA; 5 Hospital General Universitario Gregorio Marañon, Madrid, Spain; 6 H. Lee Moffitt Cancer Center and Research Institute, Tampa, FL USA; 7 Karmanos Cancer Institute, Detroit, MI USA; 8 Massachusetts General Hospital, Boston, MA USA; 9 Taussig Cancer Institute, Cleveland Clinic, Cleveland, OH USA; 10 Hopital Saint Louis, Paris, France; 11 Mayo Clinic, Rochester, MN USA; 12 Hospital Universitario Fundacion Jimenez Diaz- IISFJD-UAM, Madrid, Spain; 13 Clinica Universidad Navarra, Pamplona, Spain; 14 University of North Carolina at Chapel Hill, Chapel Hill, NC USA; 15 Fred Hutchinson Cancer Research Center, Seattle, WA USA; 16 Tennessee Oncology/Sarah Cannon Research Institute, Nashville, TN USA; 17 Mayo Clinic, Jacksonville, FL USA; 18 University of Texas MD Anderson Cancer Center, Houston, TX USA; 19 Colorado Blood Cancer Institute, Denver, CO USA; 20 Beth Israel Deaconess Medical Center, Harvard Medical School, Boston, MA USA; 21 Gustave Roussy Cancer Center, Villejuif, France; 22 Hospital Universitario Sanchinarro, Madrid, Spain; 23 Hannover Medical School, Hannover, Germany; 24 Roswell Park Comprehensive Cancer Center, Buffalo, NY USA; 25 University of Miami, Sylvester Comprehensive Cancer Center, Miami, FL USA; 26 H3 Biomedicine, Cambridge, MA USA; 27 Eisai, Inc, Woodcliff Lake, NJ USA; 28 University Hospital Leipzig, Clinic for Hematology, Cellular Therapy and Hemostaseology, Leipzig, Germany

**Keywords:** Health sciences, Medical research, Drug development

## Abstract

We conducted a phase I clinical trial of H3B-8800, an oral small molecule that binds Splicing Factor 3B1 (SF3B1), in patients with MDS, CMML, or AML. Among 84 enrolled patients (42 MDS, 4 CMML and 38 AML), 62 were red blood cell (RBC) transfusion dependent at study entry. Dose escalation cohorts examined two once-daily dosing regimens: schedule I (5 days on/9 days off, range of doses studied 1–40 mg, *n* = 65) and schedule II (21 days on/7 days off, 7–20 mg, *n* = 19); 27 patients received treatment for ≥180 days. The most common treatment-related, treatment-emergent adverse events included diarrhea, nausea, fatigue, and vomiting. No complete or partial responses meeting IWG criteria were observed; however, RBC transfusion free intervals >56 days were observed in nine patients who were transfusion dependent at study entry (15%). Of 15 MDS patients with missense SF3B1 mutations, five experienced RBC transfusion independence (TI). Elevated pre-treatment expression of aberrant transcripts of *Transmembrane Protein 14C* (*TMEM14C*), an SF3B1 splicing target encoding a mitochondrial porphyrin transporter, was observed in MDS patients experiencing RBC TI. In summary, H3B-8800 treatment was associated with mostly low-grade TAEs and induced RBC TI in a biomarker-defined subset of MDS.

## Introduction

Somatic mutations in components of the RNA spliceosome are highly recurrent in myeloid neoplasia, representing the most common class of acquired mutations in patients with myelodysplastic syndromes (MDS), chronic myelomonocytic leukemia (CMML), and secondary acute myeloid leukemia (AML) arising from MDS [1–3]. The most frequently mutated spliceosome-associated genes in hematological neoplasms include *SF3B1*, *SRSF2*, *U2AF1*, and *ZRSR2* [4–8]. The precise mechanisms by which these mutations confer a clonal advantage to mutant hematopoietic stem and progenitor cells are not completely understood, but collectively such mutations result in diverse alternative and aberrant mRNA splicing changes [9–12].

Novel therapies for MDS, AML, and CMML are needed, as many patients diagnosed with these malignancies die of complications of their disease and experience cytopenias, including transfusion-dependent anemia, that impair the patients’ quality of life [13–16]. Altered splicing is an attractive target for novel therapies, given the high frequency with which spliceosome-associated mutations are seen in myeloid neoplasms [17–19]. In addition, aberrant splicing of genes involved in heme metabolism, such as *TMEM14C, PPOX*, and *ABCB7*, has been observed in MDS patients, particularly in those carrying SF3B1 mutations, suggesting that correction of splicing defects could potentially reverse defective erythropoiesis in these patients [32].

H3B-8800 is an orally bioavailable macrocyclic lactone small molecule that binds to the SF3b complex and modulates splicing [20]. In preclinical models, including xenograft leukemia models with or without core spliceosome mutations, H3B-8800 has broad antitumor activity [21, 22]. We therefore conducted a phase I clinical trial of H3B-8800 in adult patients with MDS, CMML, and AML, both in patients harboring splicing factor mutations and in those with wild-type proteins, and report its results here.

## Methods

### Patients

The eligibility criteria were disease-specific and are summarized in Table S1. In addition, patients had to be ≥18 years old, have an Eastern Cooperative Oncology Group (ECOG) performance status of 0–2, and adequate organ function, defined as creatinine ≤1.7 mg/dL or calculated creatinine clearance (Cockroft–Gault formula) ≥50 mL/min, direct bilirubin ≤1.5 times the institution’s upper limit of normal (ULN), alanine aminotransferase and aspartate aminotransferase ≤3.0 x ULN, and albumin ≥2.5 mg/dL. MDS patients were enrolled independently of risk category [23]. Patients were also not required to have a splicing mutation to be eligible.

### Study design

Patients were enrolled using a conventional 3 + 3 dose escalation phase I design, with escalations based on a modified Fibonacci sequence scheme [24]. Dose escalations continued until a dose level at which ≥2 patients of 3–6 enrolled experienced a dose-limiting toxicity (DLT), and maximally tolerated dose (MTD) was defined as the highest dose at which no more than one out of six patients experienced a DLT. DLTs were defined as any of the following: any Grade 3 or greater study drug-related nonhematologic toxicity except for Grade 3 nausea, vomiting, fatigue, or diarrhea that resolved to Grade 1 or less within 1 week; failure to administer at least 70% of the protocol-specific dose; or prolonged myelosuppression with the persistence of Grade 4 cytopenia in the absence of persistent leukemia or blast increase 21 or more days after suspending dosing. DLTs were assessed during the first 28 days using National Cancer Institute (NCI) Common Toxicity Criteria for Adverse Events (CTCAE) version 4.03.

The starting dose of 1 mg per day on a 5-day-on, 9-day-off schedule (schedule I) was based on nonhuman primate experience; the DLT in that model system was gastrointestinal distress and colitis. In addition, a second schedule (schedule II) of 21 days on therapy and a rest of 7 days without therapy was explored. The protocol was originally designed to evaluate schedule I based on preclinical data suggesting the activity of H3B-8800 when administered intermittently. However, when limited clinical activity was observed on schedule I, schedule II was introduced to test whether more prolonged spliceosome modulation would lead to higher clinical activity. Each treatment cycle was 28 days in length. Patients could continue treatment until disease progression or development of unacceptable toxicity. Intra-patient dose escalation was permitted at Cycle 4 and beyond to dose levels demonstrated to be safe in other subsequently enrolled patients, but patients had to maintain their original dosing schedule.

The study protocol was approved by the Institutional Review Boards at each participating center; all patients provided written informed consent prior to any study screening procedures, and the study was conducted in accordance with the Declaration of Helsinki and Good Clinical Practice regulations. The study was registered at Clinicaltrials.gov (Identifier NCT02841540) prior to enrollment of the first patient. Study drug was provided by H3 Biomedicine Inc., Cambridge, Massachusetts, USA, which also provided scientific, logistical and financial support for the trial.

### Study assessments

The primary endpoints measured included occurrence of DLTs, the type and frequency of treatment-emergent adverse events (TEAEs), and serious adverse events (SAEs). Key secondary endpoints included the drug pharmacokinetics (PK) and preliminary antitumor activity, such as the overall response rate (ORR), effect of drug therapy on transfusion requirements, and overall survival (OS). Biomarker analyses and drug pharmacodynamics (PD) were included as exploratory endpoints.

### Ophthalmic safety

Because visual loss was observed in patients treated in a prior phase 1 trial of a pladienolide derivative (E7107) with chemical similarity to H3B-8800 [25, 26], and because germline mutation of the minor splicing factor PRPF8 and other related splicing factors is associated with retinitis pigmentosa [27], a detailed ophthalmologic safety plan was built into the study. Eligibility criteria included normal vitamin A levels and visual acuity that was corrected to 20/40 unless a cataract was present. Ophthalmologic evaluation, including fundoscopic imaging and visual evoked potentials, was performed during study screening and periodically throughout the duration of the trial.

### Response assessment

Clinical responses were assessed using the 2006 International Working Group (IWG) response criteria for MDS [28], the 2003 IWG criteria for AML, and the 2015 international consortium proposal of uniform response criteria for myelodysplastic/myeloproliferative neoplasms (MDS/MPN) for CMML [29]. Peripheral blood sampling, bone marrow aspirates, bone marrow biopsies, marrow cytogenetics, and cellular composition by flow cytometry were performed at the time of screening, and after Cycles 1, 2, and 4. Beyond Cycle 4, bone marrow aspirates were performed as clinically indicated based on changes in peripheral blood count, or when it was needed to establish either complete response or disease progression.

### Pharmacokinetics, pharmacodynamics, and biomarker analyses

Plasma samples for PK analyses were collected during Cycle 1 on Day 1 and Day 4 (pre-dose, and 0.5, 1, 2, 4, 6, 8, 10, and 24 h post-dose), and pre-dose and 4 h post-dose at Cycle 1 Day 15. PK analyses were conducted using Phoenix^®^ WinNonlin. For pharmacodynamic and biomarker analyses, peripheral blood samples were collected into PAXgene^®^ Blood RNA Tubes (BD Biosciences, San Jose, California) at Cycle 1 Day 1 (pre-dose, and 1, 2, 4, 10 and 24 h post-dose) for all patients. RNA was extracted using Maxwell^®^ simplyRNA Blood Kit (Promega, Madison, Wisconsin). Target engagement (i.e., splicing modulation) was measured by assessing the relative expression of representative pre- and mature-mRNA or aberrant and canonical transcripts at post-dose time points comparing to pre-dose, using a customized Nanostring nCounter gene expression panel (NanoString Technologies, Seattle, Washington). For splicing mutation analysis, peripheral blood was collected into PAXgene^®^ Blood DNA Tubes (BD Biosciences, San Jose, California). Baseline splicing mutations were identified using the Focus:Myeloid^TM^ Next Generation Sequencing (NGS) panel determined centrally by Cancer Genetics Inc. (Rutherford, NJ). Biomarker analyses were conducted using one-way ANOVA and MedCalc^®^ software. Receiver-operating-characteristics (ROC) curves were performed according to the methodology described by Hanley and McNeil, 1982 [30, 31].

## Results

### Enrolled patients

A total of 84 patients were enrolled at 26 participating centers in the United States and Europe between October 2016 and December 2018. Of the enrolled patients, 65 were treated on schedule I and 19 on schedule II. The smaller number of patients on schedule II reflects a later addition of this schedule as a protocol amendment after seven dose levels on schedule I (Fig. S1). Baseline characteristics of the enrolled patients are summarized in Table 1. Among the enrolled patients, 38 patients had AML, 4 had CMML, 20 had IPSS higher-risk MDS, and 21 had lower-risk MDS. A normal karyotype was observed in 23 MDS patients and chromosome 8 trisomy, 7q deletion, 20q deletion, and 5q deletion were observed, respectively, in five, four, four, and two patients. Nine patients had other chromosomal abnormalities and two patients had a complex (three or more abnormalities) karyotype. For 1 MDS patient, karyotyping failed, so IPSS could not be calculated. AML with myelodysplasia-related changes was the most common AML diagnosis (*N* = 20) whereas refractory anemia with excess blast was for MDS (*N* = 21). CMML1/CMML2 diagnosis was not specified. The median number and range of prior regimens were 2 (1–9), 2 (2–5) and 2 (1–4) for AML, CMML and MDS patients, respectively. A total of 62 patients were reported to be RBC transfusion dependent in the 8 weeks prior to study entry.

**Table 1:** Baseline characteristics of enrolled patients.

Characteristic	*n* (%)
Median age (range), years	74 (46–87)
Male, *n* (%)	61 (73)
Race, *n* (%)	
White	72 (86)
Black or African American	2 (2)
Asian	2 (2)
Other or missing	8 (10)
ECOG performance status, *n* (%)	
0	18 (21)
1	59 (70)
2	7 (8)
Previous anticancer regimens, *n* (%)	
0	3 (4)
1	33 (39.3)
2	24 (29)
≥3	24 (29)
Prior anticancer regimens, median	2
Prior HMA treatment, *n* (%)	73 (87)
Transfusion dependence,^a^ *n* (%)	
RBC	62 (71)
Platelet	34 (41)
Disease	
*MDS*	42 (50)^b^
Lower risk	21 (25)
Higher risk	20 (24)
IPSS missing	1 (1)
*CMML*	4 (5)
Lower risk	1 (1)
Higher risk	3 (4)
*AML*	38 (45)

*AML* acute myeloid leukemia, *CMML* chronic myelomonocytic leukemia, *ECOG* Eastern Cooperative Oncology Group, *HMA* hypomethylating agent, *IPSS* International Prognostic Scoring System, *MDS* myelodysplastic syndrome, *RBC* red blood cell.

^a^Assessed using International Working Group criteria [23].

^b^One patient had a diagnosis of RARS-T (MDS/MPN overlap syndrome).

**Fig. 1:**
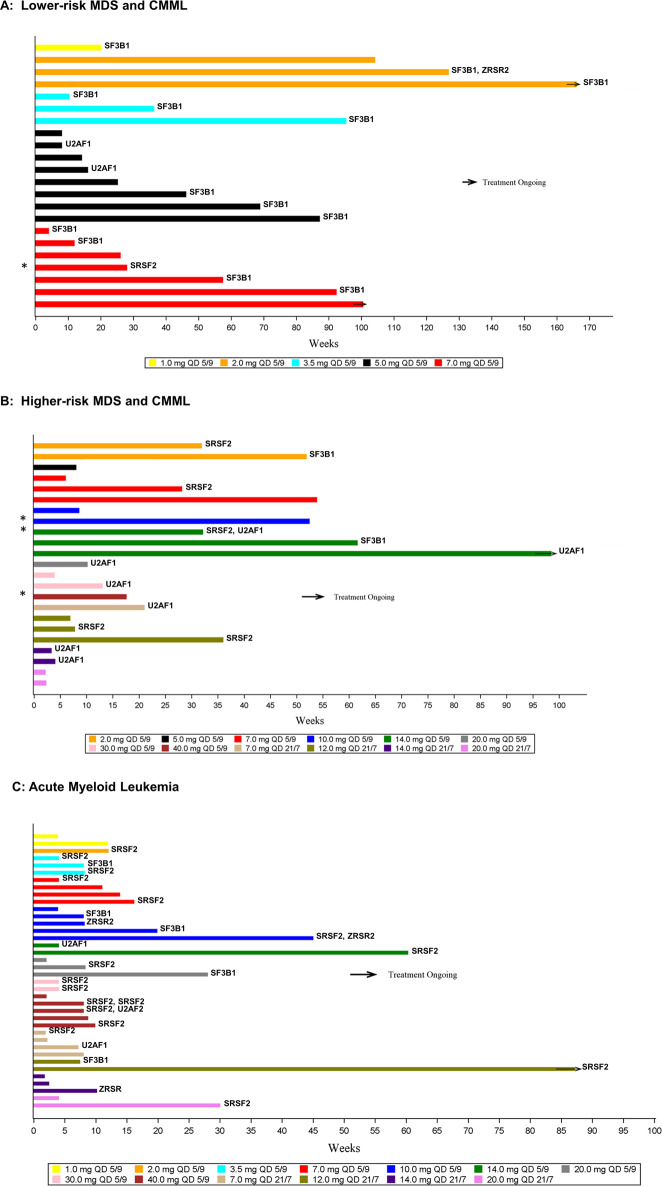
Swimmer plot of enrolled patients and their duration of therapy by disease subtype and spliceosome missense mutation at baseline. **A** Lower-risk MDS or CMML, **B** higher-risk MDS or CMML, and **C** AML. Colors designate the dose level of enrolled patients. *CMML patient. AML acute myeloid leukemia, CMML chronic myelomonocytic leukemia, MDS myelodysplastic syndrome, QD once daily.

Dose levels administered ranged from 1 to 40 mg on schedule I and from 7 to 20 mg on schedule II (Figs. 1, S1). Enrollment of lower-risk MDS patients in dose escalation was suspended after a patient with SF3B1-mutant lower-risk MDS developed pancytopenia and marrow aplasia during the first week of study therapy at the 7 mg dose level (schedule I). Thereafter, only AML, higher-risk MDS, and CMML patients were enrolled in the dose escalation portion of the trial.

### Adverse events and dose-limiting toxicities

As of April 2020, the most common treatment-related, treatment emergent adverse events (TEAEs, as judged by the investigator, ≥10% frequency) in the patients treated on schedule I were diarrhea (42%), nausea (28%), fatigue (17%), and vomiting (14%) (Table 2). The most common treatment-related TEAEs in the patients treated on schedule II were diarrhea (42%), vomiting (21%), QTcF (Fridericia method) prolongation (16%), nausea (16%), dysgeusia (11%), fatigue (11%), and hypophosphatemia (11%) (Table 2). Table 3 summarizes the most common Grade 3 and 4 events. One event of Grade 3 ocular papilledema was reported, without loss in visual acuity.

**Table 2:** Common treatment-related TEAEs (all grades) reported in ≥10% of patients by dosing schedule.

Preferred term	Dose (QD), *n* (%)								
	1, 2, 3.5, & 5 mg	7 mg	10 mg	12 mg	14 mg	20 mg	30 mg	40 mg	Total
*Schedule I*	*N* = 25	*N* = 14	*N* = 7	*N* = 0	*N* = 5	*N* = 4	*N* = 4	*N* = 6	*N* = 65
Any TEAE	19 (76)	11 (79)	3 (43)	–	5 (100)	3 (75)	4 (100)	5 (83)	50 (77)
Diarrhea	7 (28)	4 (29)	2 (29)	–	5 (100)	1 (25)	3 (75)	5 (83)	27 (42)
Nausea	3 (12)	4 (29)	2 (29)	–	1 (20)	2 (50)	3 (75)	3 (50)	18 (28)
Fatigue	3 (12)	2 (14)	1 (14)	–	0	2 (50)	1 (25)	2 (33)	11 (17)
Vomiting	1 (4)	2 (14)	2 (29)	–	1 (20)	0	0	3 (50)	9 (14)

*Schedule II*	*N* = 0	*N* = 5	*N* = 0	*N* = 5	*N* = 5	*N* = 4	*N* = 0	*N* = 0	*N* = 19
Any TEAE	–	1 (20)	–	4 (80)	4 (80)	4 (100)	–	–	13 (68)
Diarrhea	–	1 (20)	–	1 (20)	2 (40)	4 (100)	–	–	8 (42)
Vomiting	–	0	–	0	1 (20)	3 (75)	–	–	4 (21)
ECG QTcF prolonged	–	0	–	0	1 (20)	2 (50)	–	–	3 (16)
Nausea	–	0	–	1 (20)	0	2 (50)	–	–	3 (16)
Dysgeusia	–	0	–	1 (20)	0	1 (25)	–	–	2 (11)
Fatigue	–	0	–	1 (20)	0	1 (25)	–	–	2 (11)
Hypophosphatemia	–	0	–	2 (40)	0	0	–	–	2 (11)

**Table 3:** Grade 3 or 4 treatment-related TEAEs reported in >2% of patients by dosing schedule.

Preferred term	Schedule I, *n* (%) (*N* = 65)	Schedule II, *n* (%) (*N* = 19)
Anemia	4 (6)	0
ECG QTcF prolonged	2 (3)	1 (5)
Fatigue	2 (3)	1 (5)
Platelet count decreased	2 (3)	1 (5)
Hypophosphatemia	0	1 (5)
Nausea	0	1 (5)
Sinus bradycardia	0	1 (5)

The severity of TEAEs was graded using NCI-CTCAE (version 4.03).

*ECG* electrocardiogram, *NCI-CTCAE* National Cancer Institute Common Terminology for Clinical Adverse Events, *TEAE* treatment-emergent adverse event.

Altogether, six patients experienced an AE assessed by investigators as a (Cycle 1) DLT. On schedule I, one patient with lower-risk MDS and *SF3B1* mutation developed marrow aplasia at the 7 mg dose level, and two out of six patients at the 40 mg dose level were assessed by an investigator to have prolonged QTcF >500 ms. On schedule II, one of five patients treated at the 14 mg dose level experienced grade 3 sinus bradycardia, and two of four patients treated at the 20 mg dose level experienced DLTs: Grade 3 QTc prolongation in one and Grade 3 nausea that did not promptly resolve in the other. Based on these results, 40 mg in schedule I and 20 mg in schedule II appeared to exceed the DLT threshold, and the MTD was initially defined as 30 mg for schedule I (cumulative dose 300 mg H3B-8800 in 28-day cycles) and 14 mg for schedule II (294 mg H3B-8800 in 28-day cycles), and further dose escalation was stopped. An ad hoc independent cardiology review of patient ECGs was then undertaken in order to better understand the potential for cardiovascular effects with H3B-8800. Through this independent review, two of the QTc prolongation events reported as DLTs at 40 mg in schedule I and 20 mg in schedule II could not be confirmed, and the additional event at 40 mg was reported as potentially related to concomitant medications. Consequently, while the maximum tolerated dose (MTD) was not formally confirmed for either schedule I or schedule II, taking into account the totality of the data, the recommended H3B-8800 QD dose for future studies was defined as 30 mg per day on schedule I and 14 mg per day on schedule II.

### Duration of treatment

Patients remained on treatment from 12 to 1162 days; 27 patients (32%) had time on treatment greater than 180 days, 18% greater than 1 year, and 2% greater than 2 years (Fig. 1). The median duration of therapy for lower-risk MDS/CMML was 32.2 weeks, for higher-risk MDS/ CMML was 13.0 weeks, and for AML patients was 8.0 weeks, reflecting the natural history of each disease.

### Pharmacokinetics

**Fig. 2:**
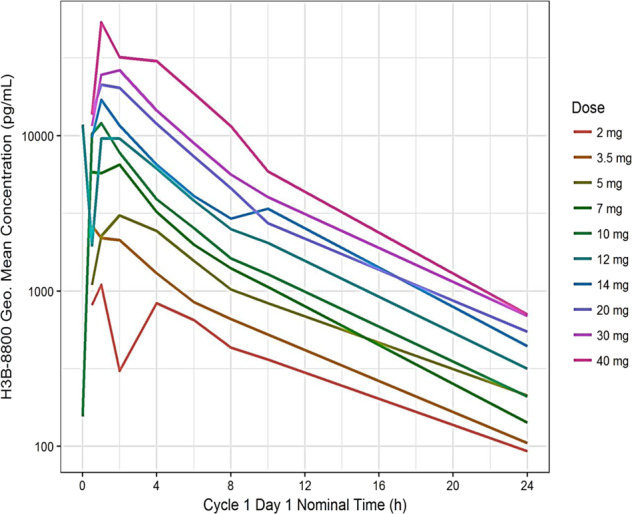
H3B-8800 pharmacokinetics. Plasma concentration in ng/mL of H3B-8800 on Cycle 1 Day 4 (depicted) for schedule I. h hours.

Preliminary PK analysis indicated that H3B-8800 is rapidly absorbed and exhibits dose-proportional increase in plasma exposure (Fig. 2). Key PK parameters via noncompartment analysis are listed in Table 4. A similar maximum cumulative dose per cycle was evaluated in both schedules (400 mg per 28 day cycle for schedule I; 420 mg per 28 day cycle for schedule II). The potential effect of covariates (including body weight and sex) on H3B-8800 plasma PK was not evaluated due to the limited number of patients enrolled at each dose level on the two schedules; however, the overall variability in PK parameters was moderate, within a range typically observed in cancer patients, without obvious outliers (Table 4). A preliminary population PK modeling estimated the inter-individual variability on clearance/bioavailability was about 34% (not shown), suggesting that the potential covariate effect, if any, would likely not be meaningful, or that the currently available data are likely not sufficient to identify a meaningful covariate effect.

**Table 4:** Summary of H3B-8800 plasma PK parameters on Day 4 following once-daily dose regiments.

Statistical summary of plasma PK parameters on Day 1					
Dose (mg)	*n*	Tmax (h)	Cmax (µg/L)	AUC0-24 (µg*h/L)	*t*1/2 (h)
1.0	3	2 (0.5–2)	0.835 (90.8)	4.89 (38.6)	5.1 (65.3)
2.0	7	2 (0.5–6)	1.09 (64.4)	8.39 (42.0)	5.4 (31.9)
3.5	6	1.5 (0.5–2)	2.78 (82.4)	15.6 (52.5)	5.4 (15.2)
5.0	9	1 (0.5–4)	3.94 (30.0)	24.5 (24.2)	5.8 (27.1)
7.0	19	1 (0.5–4)	9.61 (69.7)	39.4 (38.7)	5.3 (23.8)
10	7	1 (0.5–2)	12.7 (36.3)	51.0 (20.5)	5.5 (14.2)
12	5	2 (1–2)	12.2 (37.4)	64.7 (33.4)	5.4 (17.3)
14	10	1 (0.5–2)	25.6 (63.9)	108 (55.1)	5.1 (21.8)
20	8	0.75 (0.5–4)	33.6 (49.2)	142 (43.0)	5.1 (10.3)
30	4	1.25 (0.5–2)	34.4 (44.7)	159 (23.9)	5.3 (10.2)
40	6	0.5 (0.5–1)	73.3 (85.8)	265 (61.5)	4.5 (17.2)

Median (min–max) for Tmax and geometric mean (CV%) for other parameters.

For schedule I (doses tested ranged from 1 to 40 mg), no obvious dose dependency in the rate of treatment-related TEAEs (all grades) was observed. The incidence of these events ranged from 43 to 100% of subjects treated per dose level. At the lowest combined dose level (1, 2, 3.5, and 5 mg, *N* = 25), 76% of subjects reported a treatment-related TEAE. At the highest dose level tested (40 mg, *N* = 6), 83% of subjects reported a treatment-related TEAE. Grade 3 or higher treatment-related TEAEs were reported in subjects treated at doses ≥2 mg, with three of six subjects treated at 40 mg reporting Grade 3 AEs. For schedule II (7–20 mg), treatment-related AEs (all grades) were observed in 20% of subjects treated at the lowest dose level tested and 100% of subjects treated at the highest dose level tested, suggesting a dose dependency trend for AEs in the 21 day on/7 day off schedule. Grade 3 or higher treatment-related TEAEs were reported in dose levels ≥12 mg in schedule II, with three of the four subjects treated at 20 mg reporting Grade 3 AEs.

No obvious gender differences were observed in treatment-related AEs, although 73% of subjects enrolled were male, so the assessment of potential gender differences in the tolerability of H3B-8800 may be limited. Of the 63 subjects reporting treatment-related AEs (all grades), 45 (71%) were male, and 9 (75%) of the 12 subjects who reported Grade 3 treatment-related AEs were male.

### Clinical responses

No complete or partial responses meeting 2006 IWG criteria were observed. Based on MDS/MPN criteria, in the four subjects with a diagnosis of CMML, one complete cytogenetic remission and one clinical benefit (platelet response) were reported. No responses were observed in the remaining two CMML subjects. In addition, as of April 2020, one to six intervals of ≥56 days without RBC transfusions had been reported in nine of 62 patients (15%) who were transfusion dependent at study entry according to IWG criteria [29] (Table S2). All of these patients met the IWG criteria for a response of erythroid hematological improvement, except one who had a hemoglobin of 9.1 g/dL at baseline. All received H3B-8800 within schedule I. Eight of the nine patients had a first RBC TI that initiated in weeks 1–24 of the study. Of the nine RBC TI cases, one was observed in a CMML subject (25%) and eight in MDS (19%). The CMML patient also experienced a 21-week period of platelet transfusion independence. Four of the nine patients experienced RBC TI at the 7 mg dose. Median duration of RBC TI was 13 weeks. Median time to onset of RBC TI was 15 weeks. Two additional patients (1 AML, 1 MDS) experienced RBC TI periods of ≥56 days but their transfusion dependence prior to study entry could not be verified. One case of a 22-week platelet transfusion independence without RBC TI was also observed in a higher-risk MDS patient who received the 7 mg dose in schedule II.

### Biomarker analyses

Mutation data on key spliceosome proteins (SF3B1, SRSF2, U2AF1, and ZRSR2) were generated from PBMCs using two NGS panels in duplicate samples (Fig. 1). SF3B1 mutations were the most common ones in MDS patients (15 missense mutations in 41 patients, 36.6%), particularly in lower-risk MDS (57%) (Fig. S1). SF3B1 mutations were also observed in 5 of 38 (13%) AML patients and in none of the 4 CMML patients. A listing of patient characteristics for the subset of patients with missense SF3B1 mutations is shown in Table S3. There was insufficient sampling of PBMCs on study treatment to determine clonal changes in all of the patients who experienced RBC TI events. Of the 20 patients with missense SF3B1 mutations at study entry, 5 (25%) experienced RBC TI events. Three of the patients with RBC TI periods had a diagnosis of refractory anemia with ring sideroblasts (RARS), one of refractory anemia with excess blasts, and one of RARS with thrombocytosis. No RBC TI periods were observed in four patients with SF3B1 mutations and a diagnosis of AML.

**Fig. 3:**
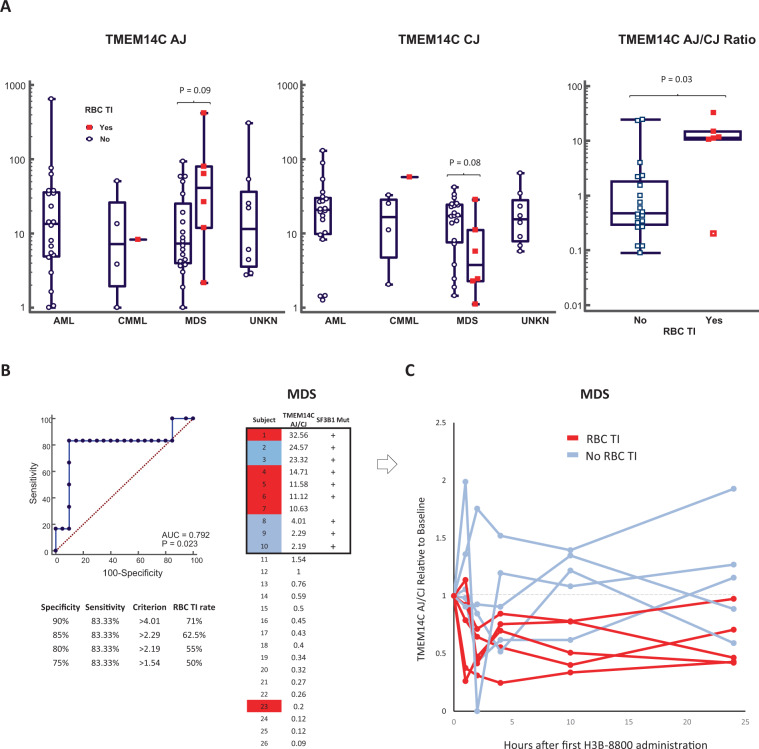
Pre-treatment *TMEM14C* AJ/CJ ratio may predict the RBC TI of MDS subjects on H3B-8800 treatment. **A** Box plots representing the relationship between pre-treatment *TMEM14C* AJ/CJ (NanoString) and RBC TI on study by tumor indication. Diagnosis of AML, MDS, or CMML is shown as per central assessment. **B** ROC curve analyses and ranking of MDS subjects with available pre-treatment *TMEM14C* AJ/CJ data. **C** *TMEM14C* AJ/CJ expression ratio post H3B-8800 treatment in MDS subjects with high pre-treatment *TMEM14C* AJ/CJ ratio. RBC TI red blood cell transfusion independence, MDS myelodysplastic syndromes.

PBMC samples for pharmacodynamics assessments were collected from 59 of the 84 patients, including 26 pre-treatment samples from MDS patients, and gene expression data generated using NanoString probes. A total of 61 splicing markers were investigated (Table S4). A general modulation of splicing markers post-dosing was observed (Fig. S2). Seven patients who experienced RBC TI > 56 days had available gene expression data. Trends for increased pre-treatment aberrant splicing junction (AJ) transcripts and decreased pre-treatment canonical splicing junction (CJ) transcripts of the gene encoding for *TMEM14C* were observed in the MDS patients who experienced RBC TI (Fig. 3A). ROC curve analyses indicated that the pre-treatment ratio of *TMEM14C* AJ/CJ was predictive of the onset of RBC TI on H3B-8800 treatment in MDS patients with an optimal Youden index *J* = 0.733 for an associated criterion of >4.01 with a 83.3% sensitivity and 90% specificity. Of the seven MDS patients with (*TMEM14C* AJ/CJ > 4.01, Fig. 3B), five experienced events of RBC TI with H3B-8800 (71%). SF3B1 mutations were detected in all but one of them (Fig. 3B). Downregulation of the *TMEM14C* AJ/CJ ratio with H3B-8800 dosing was also observed in the patients who experienced RBC TI, with a nadir at 2–10 h (Fig. 3C). Because of the potential relevance of these findings, quantification of *TMEM14C* aberrant and canonical transcripts was repeated using RT-qPCR in residual study samples (*N* = 20, including four patients who experienced RBC TI). Additional time points were also included in these experiments (Cycle 1 Day 4). Pre-treatment expression of TMEM14C AJ was higher in Cycle 1 Day 1 PBMC samples from patients who experienced RBC TI with H3B-8800 treatment (Fig. S3). Likewise, pre-treatment expression of *TMEM14C* AJ was also higher in Cycle 1 Day 4 samples from those patients (Fig. S3).

## Discussion

This study represents the first clinical trial of a targeted splicing modulator in patients with myeloid neoplasms. The observed adverse event profile was consistent with gastrointestinal disturbances observed in pre-clinical models, and reversible QTc prolongation represented the most common investigator-assessed DLT. Despite 88% of enrolled patients having splicing mutations, dose-dependent target engagement, a predictable PK profile of the study drug, and safety even with prolonged dosing in many patients, complete or partial responses were not observed. It is unclear why the clinical results did not recapitulate murine experiments that had suggested synthetic lethality and reduction of the burden of mutant cells with disruption of residual wild-type splicing [21, 22]. This outcome could indicate that the level of splicing inhibition required to achieve cell killing is higher than what was achieved in human subjects, or because abnormal splicing, although contributing to disease initiation, may no longer be required for survival in transformed cells.

The observation of RBC TI and the identification of a subset of MDS patients who could benefit from H3B-8800 treatment in terms of RBC transfusion requirements was a key finding of this study, however. Mutated SF3B1 has been associated with the ring sideroblast phenotype, which is characterized by defects in heme biosynthesis and iron accumulation in mitochondria. Three genes involved in heme biosynthesis and iron metabolism are recurrently aberrantly spliced in SF3B1-mutated patients with MDS: *ABCB7, PPOX*, and *TMEM14C* [32]. Our results suggest that H3B-8800 might be able to inhibit *TMEM14C* aberrant splicing in MDS patients. Patients with a relative excess of *TMEM14C* AJ transcripts appeared to be the most likely to benefit from H3B-8800 therapy (Fig. 3A). These preliminary results should be interpreted with caution due to the very low levels of *TMEM14C* expression observed in these experiments, however. New high-sensitivity assays would need to be developed to investigate further the dynamics of AJ and CJ transcript expression in disease and in response to H3B-8800 treatment. Furthermore, although TMEM14C is known to be required for terminal erythropoiesis [33], it has been previously reported that *TMEM14C* splicing does not modify TMEM14C protein sequence or expression [32]. Those studies, however, were conducted using expression reporter constructs in cell lines and may not accurately represent the pathobiology of MDS. Further basic research on this question should be performed.

One limitation of the present study is that the NanoString gene expression panel employed did not include molecular probes for *ABCB7* or *PPOX*. Thus, it is possible that modulation of *ABCB7* or *PPOX* could contribute to H3B-8800-induced RBC TI and should be investigated in future studies. PPOX works with TMEM14C to facilitate the mitochondrial transport of porphyrins [32]. Downregulation of the iron exporter ABCB7 has been linked to increased mitochondrial iron accumulation observed in MDS patients with ring sideroblasts, and loss of *ABCB7* expression in experimental models causes defective heme biosynthesis, mitochondrial iron overload, and apoptosis of erythroid progenitors [34]. RBC TI periods on H3B-8800 treatment were also observed in patients without SF3B1 mutations (Fig. S4), suggesting that modulation of splicing by wild-type SF3B1 protein also plays a role in the mechanism of action of this agent in some patients and deserves further investigation. Of interest, patients with SF3B1 mutations appeared to have experienced RBC TI at lower doses than those with SF3B1 wild-type status (Fig. S4).

Selected spliceosome markers showed a pattern of modulation consistent with preclinical observations, namely that maximum modulation happened approximately at 4–10 h after dosing and then diminished [21]. It is possible that higher clinical activity could require a more prolonged inhibition of aberrant splicing. The use of alternative dose regimens, for example twice-daily dosing, could contribute to more consistent splicing inhibition and a different pattern of clinical response and should be investigated.

In conclusion, treatment with H3B-8800 had an acceptable adverse event profile, predictable PK, and modulated splicing, including downregulation of aberrantly spliced *TMEM14C*. SF3B1 mutation and a high pre-treatment ratio of *TMEM14C* AJ/CJ were associated with RBC TI in MDS patients. These parameters could help in the identification or stratification of patients who may benefit from H3B-8800 treatment. Further investigation of H3B-8800 in SF3B1 mutant MDS is warranted, including the exploration of other dosing schedules.

## Supplementary information

Supplemental Materials
