# Supplementary material for: Phase I First-in-Human Dose Escalation Study of the oral SF3B1 modulator H3B-8800 in myeloid neoplasms
Source: Leukemia. 2021 Jun 25;35(12):3542–50. doi: 10.1038/s41375-021-01328-9 (PMC8632688; doi:10.1038/s41375-021-01328-9)
Supplement: Supplementary file 1 — Supplemental Materials [file 41375_2021_1328_MOESM1_ESM.docx]

**Supplemental material for:**

Steensma, D. P. et al. **Phase I First-in-Human Dose Escalation Study of the oral SF3B1 Modulator H3B-8800 in Myeloid Neoplasms**

**Supplemental Tables**

**Table S1. Disease-specific eligibility criteria**

| **Diagnosis** | **Diagnosis-Specific Eligibility Criteria** |
| --- | --- |
| **CMML** | Treated with at least one prior therapy (e.g., hydroxyurea or an HMA) |
| **Higher-risk MDS (IPSS Intermediate-2 or High)^23^** | Intolerant of HMAs in judgment of investigator/patient **or**  Not responded to 4 treatment cycles of decitabine or 6 treatment cycles of azacitidine **or**  Progressed at any point after initiation of an HMA |
| **Lower-risk MDS (IPSS Intermediate-1 or Low)^23^** | Transfusion-dependent for RBCs or platelets  RBC transfusion-dependent patients must also have been failed by ESAs or have serum erythropoietin level >500 U/L  Platelet counts above 50 × 10^9^/L in the absence of transfusion for 8 weeks |
| **AML** | Declined or not considered a candidate for intensive induction chemotherapy by the enrolling physician  Previously-treated participants should have evidence of persistent or recurrent AML in the peripheral blood and/or bone marrow that is refractory to, or has relapsed from, the most recent prior line of treatment  WBC <15 × 10^9^/L |
| AML, acute myeloid leukemia; CMML, chronic myelomonocytic leukemia; ESAs, erythropoiesis stimulating agents; HMA, hypomethylating agent; IPSS, International Prognostic Scoring System; MDS, myelodysplastic syndrome; RBC, red blood cell; WBC, white blood cell. | |

**Table S2. Subjects who experienced RBC TI >56 days**

| **Disease Type** | **IPSS** | **Age, Gender** | **Starting Dose (mg)** | **Number of RBC TI Periods** | **Longest RBC TI Period (days)** | **Time to First RBC TI (days)** |
| --- | --- | --- | --- | --- | --- | --- |
| MDS | Higher | 78,M | 7 | 1 | 107 | 104 |
| MDS/MPN† | Lower | 85,M | 2 | 1 | 56 | 111 |
| MDS | Lower | 68,M | 2 ** | 5 | 85 | 88 |
| MDS | Higher | 68,F | 2 | 1 | 121 | 126 |
| MDS | Lower | 80,M | 3.5 | 2 | 98 | 101 |
| MDS* | Lower | 69,M | 5 | 1 | 87 | 398 |
| CMML | Lower | 78,F | 7 | 1 | 66 | 4 |
| MDS | Lower | 72,F | 7 | 6 | 91 | 129 |
| MDS | Lower | 84,M | 7 | 4 | 98 | 73 |

† RARS-T diagnosis. *Baseline Hb was 9.1 g/dL.** Patient received 7 mg of H3B-8800 during his last 2 RBC TI periods.

**Table S3. Listing of Patients with Missense SF3B1 Mutations in Peripheral Blood at Cycle 1 Day 1.**

| **Age, Race, Gender** | **Diagnosis*** | **Histology*** | **SF3B1** |
| --- | --- | --- | --- |
| 58, W, M | MDS | RARS | p.K700E |
| 66, W, M | MDS | RARS | p.R625G |
| 61, W, M | MDS | RARS | p.K700E |
| 68, W, M | MDS | RARS | p.H662Q |
| 85, U, M | MDS | RARS | p.H662Q |
| 72, W, F | AML | AML with myelodysplasia | p.K700E |
| 81, W, M | MDS | RARS | p.K700E |
| 80, O, M | MDS | RARS | p.K700E |
| 69, W, M | MDS | RA | p.R625C |
| 82, W, M | AML | AML with maturation | p.K666N |
| 79, U, M | AML | AML with myelodysplasia | p.K700E |
| 71, W, M | N/E | N/E | p.K700E |
| 81, W, M | N/E | N/E | p.R625C |
| 84, W, M | MDS | RARS | p.K700E |
| 72, W, M | N/E | N/E | p.E592K |
| 76, W, M | MDS | RA | p.K700E |
| 72, W, F | AML | AML with myelodysplasia | p.R625H |
| 68, W, F | MDS | RAEB | p.R625C |
| 69, W, M | MDS | RARS | p.K700E |
| 72, W, M | MDS | RAEB | p.E622D |

* Based on central pathology assessment. Diagnosis was not confirmed in 2 MDS and 1 AML patients (N/E). O, other; W, white; M, male; U, unknown; F, female; MDS, myelodysplastic syndrome; AML, acute myeloid leukemia; N/E, non-evaluable; RARS, refractory anemia with ring sideroblasts; RA, refractory anemia.

| **Junctions that may not be associated with a particular mutation** | | | | **Junctions affected by SF3B1 mutations** | **Junctions affected by U2AF1 mutations** | **Junctions affected by SRSF2 mutations** |
| --- | --- | --- | --- | --- | --- | --- |
| COASY-mat | PLEKHJ1-mat | PWP1-mat | CDKN1A-mat | TMEM14C_CJ | EFCAB14_e6-e8 (AJ_1) | RHOT2-CJ |
| COASY-pre | PLEKHJ1-pre_1 | PWP1-pre | CDKN1A-pre | TMEM14C_AJ | EFCAB14_e7-e8 (CJ_1) | RHOT2-AJ |
| EIF4A1-mat | PLEKHJ1-pre_2 | DYNLT1-mat | OGFOD2-mat | ZDHHC16_CJ | IRAK4_e4_e5  (AJ_3) | CEP57-CJ |
| EIF4A1-pre | RBM5-mat | DYNLT1-pre | OGFOD2-pre | ZDHHC16_AJ | IRAK4_e3_e5  (CJ_3) | CEP57-AJ |
| FBXW5-mat | RBM5-pre_1 |  | TRIM65-mat | SLTM_CJ | TEX30_e5-e6  (AJ_2) |  |
| FBXW5-pre | RBM5-pre_2 |  | TRIM65-pre | SLTM_AJ | TEX30_e4-e6  (CJ_3) |  |
| MBD4-mat | ADPRHL2-mat |  | UNC50-mat | SNURF_CJ | UBA2_e1-e3  (AJ_4) |  |
| MBD4-pre | ADPRHL2-pre |  | UNC50-pre | SNURF_AJ | UBA2_e1-e2  (CJ_1) |  |
| SLC25A19-mat  (e3-4) | ADPRHL2-pre_2 |  | KRI1-mat | ZNF561_AJ | WSB1_Intron-e6  (AJ_2) |  |
| SLC25A19-mat  (e5-6) | CDK9-mat |  | KRI1-pre | TAK1_CJ | WSB1_e6  (CJ_1) |  |
| DPH2-mat  (e2-3) | CDK9-pre |  |  | ZNF410_CJ |  |  |

**Table S4. Splicing markers**

**Supplemental Figure Legends**

**Figure S1. Study Design Schema.** a: ClinicalTrials.gov Identifier: NCT02841540.

b: Creatinine ≤1.7 mg/dL or calculated CrCl ≥50 mL/min; direct bilirubin ≤1.5 x ULN; AST/ALT ≤3.0 x ULN; albumin ≥2.5 mg/dL; normal vitamin A; vision corrects to 20/40 unless due to cataracts. c: Administered once daily, on a 28-day cycle. ALT, alanine aminotransferase; AML, acute myeloid leukemia; AST, aspartate aminotransferase; CMML, chronic myelomonocytic leukemia; CrCl, creatinine clearance; MDS, myelodysplastic syndrome; ULN, upper limit normal.

**Figure S2. H3B-8800 Modulates Splicing.** Heat map showing an H3B-8800 dose-dependent modulation of the relative expression of splicing markers. Patient numbers from left to right were 6, 6, 9, 8, 3, 7, 5, 3, 5, 4, 4, 3, 6. ES, exon skipping.

**Figure S3. RT-qPCR Gene Expression in Residual Samples.** Box plots representing gene expression by RT-qPCR for patient subsets as shown in the figure. Kruskal Wallis test was used to determine differences between groups. p values for RBC TI yes vs. no were 0.055 and 0.025 for the TMEM14C AJ/CJ ratio at Cycle 1 Day 1 and Cycle 1 Day 4, respectively. RT-qPCR, quantitative reverse transcription PCR.

**Figure S4. Mutations in patients experiencing RBC TI.** Summary of mutations detected pre-treatment on Cycle 1 Day 1 in peripheral blood of patients who experienced RBC TI periods with H3B-8800 treatment.

**Figure S1**


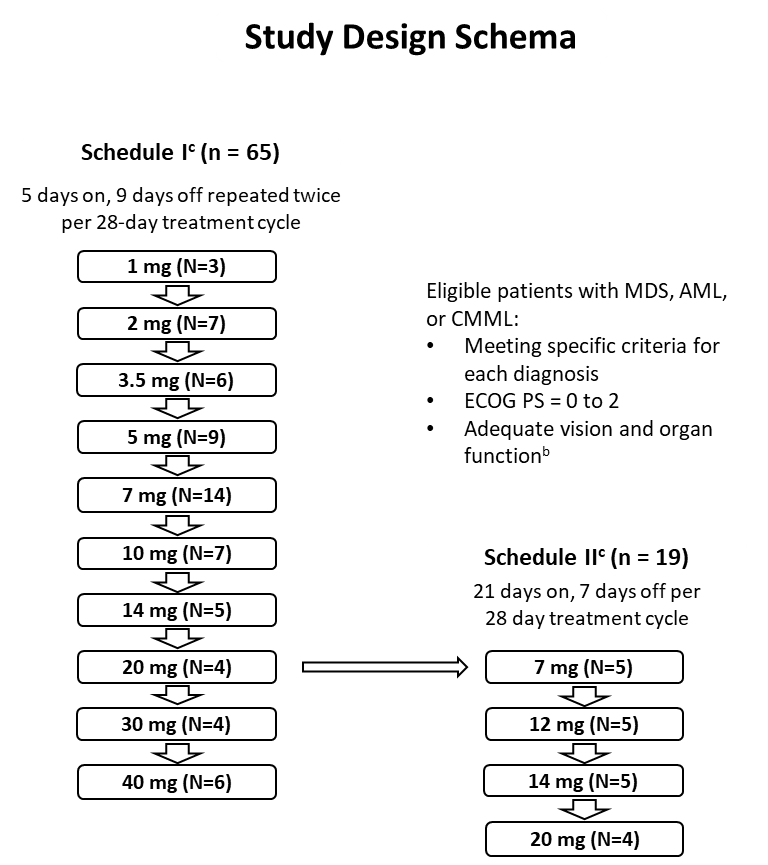


**Figure S2**

**
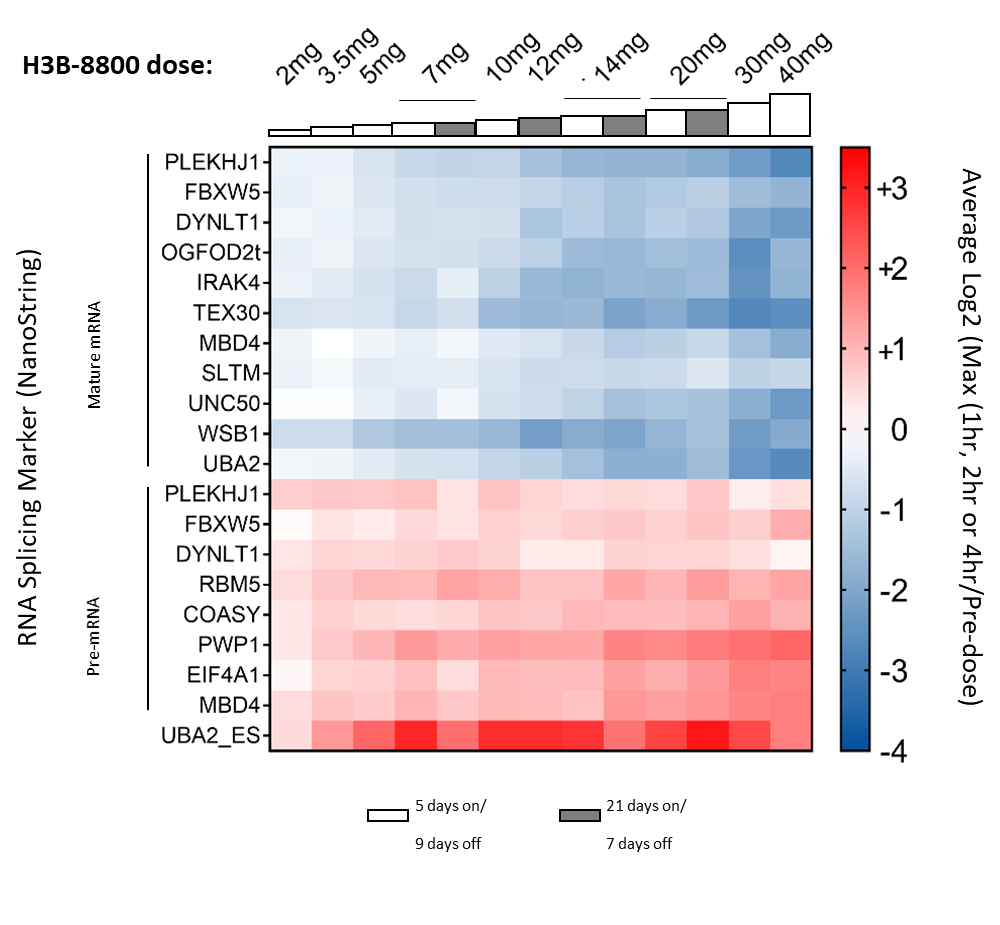
**

**Figure S3**

**
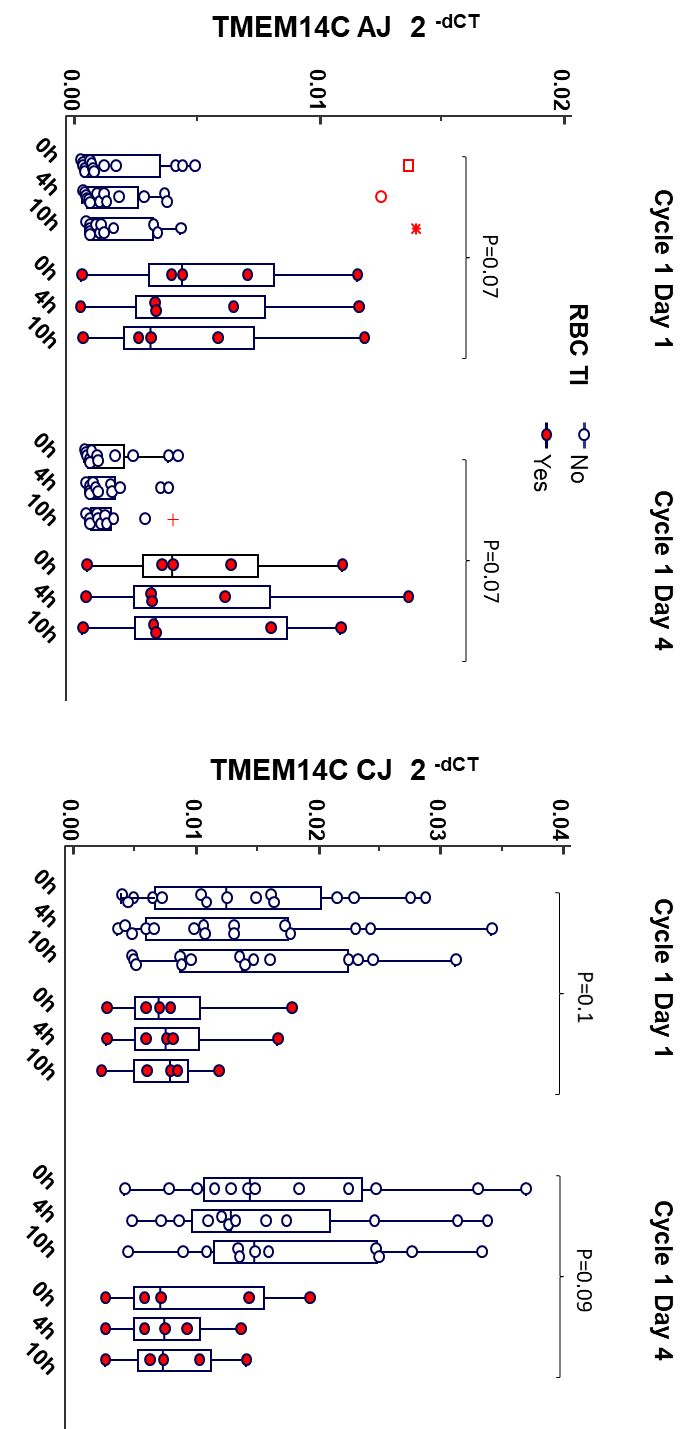
**

**Figure S4**

**
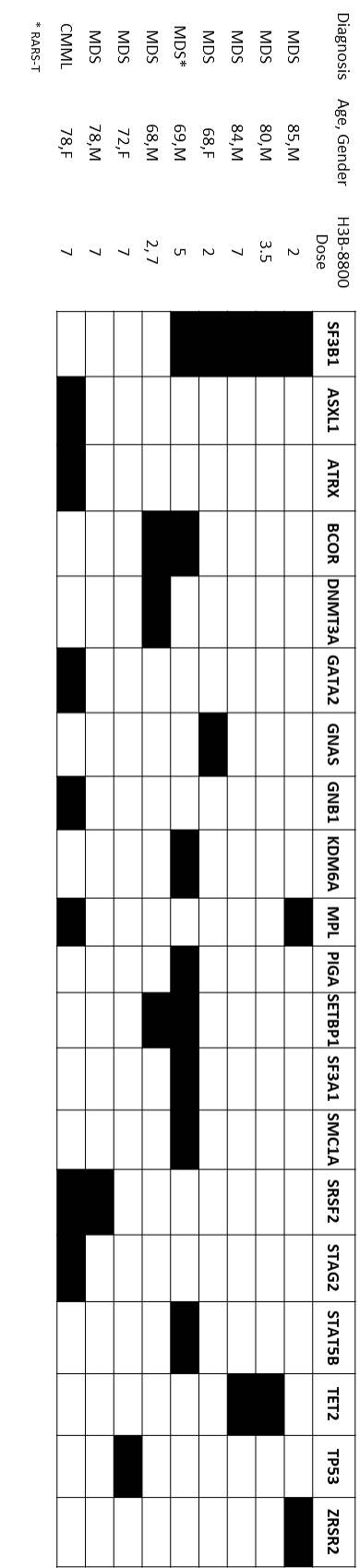
**
